# Zoonotic malaria transmission and land use change in Southeast Asia: what is known about the vectors

**DOI:** 10.1186/s12936-022-04129-2

**Published:** 2022-03-31

**Authors:** Bram van de Straat, Boni Sebayang, Matthew J. Grigg, Kyran Staunton, Triwibowo Ambar Garjito, Indra Vythilingam, Tanya L. Russell, Thomas R. Burkot

**Affiliations:** 1 Australian Institute of Tropical Health and Medicine, James Cook University, Cairns, Australia; 2 Menzies School of Health Research & Charles Darwin University, Casuarina, Australia; 3 Institute for Vector and Reservoir Control Research and Development, National Institute of Health Research and Development (NIHRD), The Ministry of Health of Indonesia, Jakarta, Indonesia; 4 Department of Parasitology, Faculty of Medicine, Universiti Malaya, Kuala Lumpur, Malaysia

**Keywords:** Zoonotic malaria, *Plasmodium knowlesi*, Leucosphyrus Group, Mosquito vectors, Vector behaviour, Human land-use

## Abstract

Zoonotic *Plasmodium* infections in humans in many Southeast Asian countries have been increasing, including in countries approaching elimination of human-only malaria transmission. Most simian malarias in humans are caused by *Plasmodium knowlesi*, but recent research shows that humans are at risk of many different simian *Plasmodium* species. In Southeast Asia, simian *Plasmodium* species are mainly transmitted by mosquitoes in the *Anopheles leucosphyrus* and *Anopheles dirus* complexes. Although there is some evidence of species outside the Leucosphyrus Group transmitting simian *Plasmodium* species, these await confirmation of transmission to humans. The vectors of monkey malarias are mostly found in forests and forest fringes, where they readily bite long-tailed and pig-tailed macaques (the natural reservoir hosts) and humans. How changing land-uses influence zoonotic malaria vectors is still poorly understood. Fragmentation of forests from logging, agriculture and other human activities is associated with increased zoonotic *Plasmodium* vector exposure. This is thought to occur through altered macaque and mosquito distributions and behaviours, and importantly, increased proximity of humans, macaques, and mosquito vectors. Underlying the increase in vector densities is the issue that the land-use change and human activities create more oviposition sites and, in correlation, increases availably of human blood hosts. The current understanding of zoonotic malaria vector species is largely based on a small number of studies in geographically restricted areas. What is known about the vectors is limited: the data is strongest for distribution and density with only weak evidence for a limited number of species in the Leucosphyrus Group for resting habits, insecticide resistance, blood feeding habits and larval habitats. More data are needed on vector diversity and bionomics in additional geographic areas to understand both the impacts on transmission of anthropogenic land-use change and how this significant disease in humans might be controlled.

## Background

Since the turn of the millennium, substantial progress has been made to reduce the global incidence of human malaria caused by *Plasmodium falciparum* and *Plasmodium vivax*. Indeed, elimination of *P. falciparum* and *P. vivax* has been achieved in a number of countries [1]. Largely responsible for this success has been the wide-scale use of insecticide-treated nets (ITNs) and indoor residual spraying (IRS), coupled with improved point-of-care diagnostics and treatment with artemisinin-based combination therapy. Recently, the number of reported zoonotic *Plasmodium* species infections in humans have been increasing, including in countries that have eliminated human malaria (Singapore, Brunei) as well as in countries such as Malaysia, which is close to eliminating human-only malaria transmission [2–4].

The most common cause of zoonotic malaria in Southeast Asia is due to the natural macaque host parasite *Plasmodium knowlesi*. The textbook ‘*The Primate Malarias’* [5] provides an excellent review of the discovery, in 1932, and subsequent transmission experiments of *P. knowlesi* to humans [6]. Due to the discovery of a naturally-transmitted *P. knowlesi* case [7], *The Primate Malarias* presciently warned that *P. knowlesi* could form a potential threat to humans as a zoonosis. However, it was thought at the time that zoonotic cases of *P. knowlesi* (and other simian malarias) were rare due to the strongly sylvan nature of both its primate hosts and mosquito vectors. Now, fifty years after the publication of *The Primate Malarias*, the understanding of how human activities are affecting, and potentially facilitating, the natural transmission of ‘monkey malaria’ to man is only just beginning.

Natural transmission of *P. knowlesi* to humans on a large scale was first described in 2004 in Sarawak, East Malaysia [8]. Cases have since been discovered throughout the Southeast Asian region, including Indonesia [9, 10], Lao PDR [11], Malaysia [12, 13], Myanmar [14], the Philippines [15], Singapore [16, 17], Brunei [18], Cambodia [19], Thailand [20] and Vietnam [21]. Indeed, while human malaria transmission is waning, zoonotic *Plasmodium* infections in humans are rising and *P. knowlesi* malaria is now the dominant malaria in humans in Malaysia [4, 22]. *Plasmodium knowlesi* is primarily a parasite of non-human primates, especially of long-tailed macaques, and northern and southern pig-tailed macaques (*Macaca fascicularis*, *Macaca leonina*, and *Macaca nemestrina,* respectively). Human-to-human transmission via a mosquito vector, demonstrated experimentally, cannot be excluded as occurring in nature [7, 23]. Additionally, the detection of *Plasmodium inui*, *Plasmodium inui*-like, *Plasmodium cynomolgi*, *Plasmodium knowlesi*, and *Plasmodium coatneyi* parasites in blood samples from Malaysia [24] and recent reports from both Peninsular Malaysia [25] and Malaysian Borneo [26, 27] show naturally-acquired human infections of several of the most common *Plasmodium* species in mosquitoes and macaques in Southeast Asia. The emergence of novel zoonotic malarias will complicate malaria control in the region.

This review will focus on transmission of *P. knowlesi* to humans in Southeast Asia, as such infections account for the highest incidence of zoonotic malaria and is the species for which relatively more is known about the transmission and vectors. The influence of anthropogenic land-use changes on the distributions and behaviours of the vectors of *P. knowlesi* malaria in Southeast Asia, with consequent spill-over transmission to humans, will be highlighted.

## Vectors of zoonotic malaria in Southeast Asia

Tables 1 and 2 summarize what is known and the strength of the evidence for vector behaviour and transmission indicators [28] for human biting species known or strongly suspected to vector *P. knowlesi* to humans. Here, vector status is defined as regards *P. knowlesi* transmission to humans as confirmed, incriminated, or suspected. Confirmed vectors are species in which *P. knowlesi* sporozoites were found in the salivary glands, incriminated vectors are species in which *P. knowlesi* DNA was identified by PCR and suspected vectors refer to confirmed vectors of other simian malarias that occur in areas of *P. knowlesi* transmission. Although *Anopheles hackeri* was found to be a potential vector of simian malaria species including *P. knowlesi* in Peninsular Malaysia [29], it was later found to be mainly (if not entirely) zoophagic and not attracted to humans [30, 31]. Also, it has been suggested that *Anopheles kochi* might act as a vector of simian malaria species in Singapore [16]. However, *An. kochi* is strongly zoophagic and bites humans only very sporadically [31–33]. Hence, both *An. hackeri* and *An. kochi* are not considered to be important vectors of *P. knowlesi* and other simian malaria species to humans and will not be discussed further in this manuscript.

**Table 1:** Vector species of *Plasmodium knowlesi*: evidence for WHO Indicators

*P. knowlesi* vector species	WHO Indicators											Literature
	*Occurrence*	*Density*	*Biting time*	*Biting location*	*HBR*	*Resting location*	*Resistance*	*Larval habitat*	*HBI*	*Sporozoite rate*	*EIR*	
*An. latens*	***	**	**	**	**	°		*	*	**	*	[34, 40, 46, 49, 50, 56, 60, 100]
*An. leucosphyrus*	**			°								[44, 60, 101]
*An. balabacensis*	***	***	***	***	**		°			**	*	[23, 40–42, 45, 49, 51, 54, 100, 102, 103]^a^
*An. introlatus*	**	**	*	**	*							[46, 53, 56, 104]
*An. dirus*	***	***	**	**	**		**	**		**	°	[21, 38, 47, 48, 52, 61, 68, 105]
*An. cracens*	**	**	**	**	*			*	°	**	*	[12, 44, 67, 106]
*An. donaldi*	**	**	*	*	*					*		[41, 42, 45, 51, 100]
*An. sundaicus*	*	*								°		[43]
*An. letifer*	*	*		°						*		[40, 56]

This table includes published evidence on biological indicators of species that are known or strongly suspected to transmit zoonotic malaria, based on the WHO indicators for vector control. Only the publications that studies a species in its role as vector for zoonotic malaria are included; i.e., An. dirus is also a vector for human malarias but only research on its role as vector for zoonotic malaria is included. Evidence of direct findings are indicated by asterisks: * weak evidence (information from a single publication, or only mentioned as a sidenote in other publications); ** medium evidence (information from less than 5 publications, of which only a minority was mentioned as sidenote); *** strong evidence (information from 5 or more publications, none of which mentioned the evidence as a sidenote). Circumstantial evidence indicated by a. *HBR* human biting rate, *HBI* human blood index, *EIR* entomological inoculation rate

^a^Some additional information on *An. balabacensis* was provided by the Ministry of Health of Indonesia. We have included this document as Reference [106], which is an official document issued by the Ministry of Health, Indonesia and is available upon request

**Table 2:** Information on vector behaviours

*P. knowlesi* vector species	WHO Indicators											Literature
	*Occurrence*	*Density (min|max)*	*HBR*	*HBI*	*Biting time*	*Biting location*	*Resting location*	*Resistance*	*Larval habitat*	*Sporozoite rate*	*EIR*	
*An. latens*	Borneo: Sarawak, South/Central/North Kalimantan	1 | 1073	Highest in forest fringe and forest	Human—monkey = 1:1.12^6^; 1.3:1^7^	Starts early, peak varies (20.00–01.00)	Outdoors	°		Freshwater pools, puddles; still, shaded water; little vegetation	0.70–1.40%	*	[34, 40, 46, 49, 50, 56, 60, 100]
*An. leucosphyrus*	Sumatra, Java		Highest in forest		Peaks between 00.00–04.00	Outdoors			Freshwater pools, shaded water, jungle pools, seepage springs, marshes, hoof prints			[34, 60, 101]
*An. balabacensis*	Malaysia: Sabah, North Sarawak; Indonesia: North/South Kalimantan, Sumatra, Java, West Nusa Tenggara	1 | 1791	Highest in forest edge and plantations		Early, peaks between 18.00–21.00	Mainly outdoor, some indoor biting present in villages		°		1.03–3.42% (100%)	*	[23, 40–42, 45, 49, 51, 100, 102–104]^a^
*An. introlatus*	Peninsular Malaysia, Sumatra	4 | 135	Highest in forest		Early, peaks between 19.00–21.00	Outdoors						[46, 53, 56, 104]
*An. dirus*	Thailand, Lao PDR, Cambodia, Vietnam, Peninsular Malaysia	8 | 5686	Highest in forest 0.3–17.4		Early peak, biting continues through the night	Outdoors, occasional indoor biting		**	Freshwater pools, puddles; still, shaded water, along slow streams	(0%), 0.54–2.0%	°	[21, 38, 47, 48, 52, 61, 68, 105]
*An. cracens*	Peninsular Malaysia	40 | 648	High in forest and fruit farm	°	Early, peaks between 19.00–21.00	Outdoors			Freshwater pools, puddles; still, shaded water; little vegetation	0.60–2.90%	*	[12, 44, 66, 104]
*An. donaldi*	Malaysia: Sarawak, Sabah,	3 | 251	High in forest		Early, peaks between 18.00–19.00	Outdoors				*		[41, 42, 45, 51, 100]
*An. sundaicus*	As possible *P. knowlesi* vector: Nicobar Islands, India	350										[43]
*An. letifer*	Malaysian Borneo	172				°				3.48%		[40, 56]

This table includes information based on published data on biological indicators of species that are known to transmit P. knowlesi malaria to humans, based on the WHO indicators for vector control. The table only includes publications that studies a species in its role as vector for zoonotic malaria; i.e., An. dirus is also a vector for human malarias but only research on its role as vector for zoonotic malaria is included; An. donaldi, An. letifer and An. sundaicus await confirmation. *HBR* human biting rate, *HBI* human blood index, *EIR* entomological inoculation rate

^a^Some additional information on *An. balabacensis* was provided by the Ministry of Health of Indonesia. We have included this document as Reference [106], which is an official document issued by the Ministry of Health, Indonesia and is available upon request

**Fig. 1:**
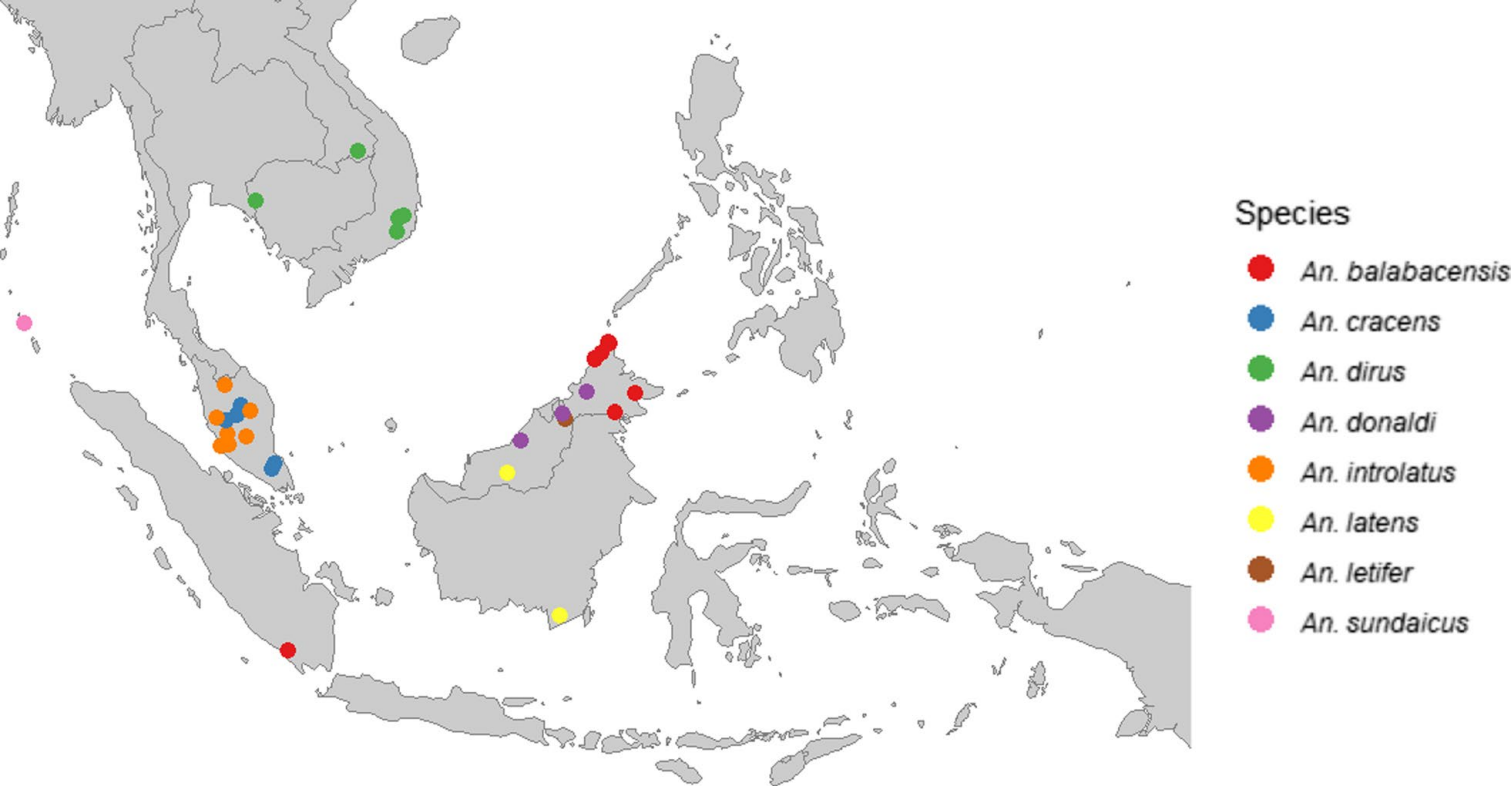
The geographical distribution of research focussing on *P. knowlesi* vectors and vector behaviours. Papers were included only when the research concerned (suspected) *P. knowlesi* or other simian malaria species transmission. Maps were made with R statistical software (R version 4.0.2), packages ‘tidyverse’ and ‘maps’

Although some indicators (occurrence, abundance, biting location) are relatively well-studied for most species, little to no evidence exists for many important indicators (resting location, larval habitats, sporozoite rates and EIR) (Table 1). What is known about the vectors’ behaviours, especially the *Anopheles leucosphyrus* complex, is based on limited knowledge from a few point sources within the geographical distribution of the Leucosphyrus Group (Fig. 1). Additionally, even for *Anopheles balabacensis* and *Anopheles latens*, the best-studied and, as thus far known, most competent vectors of *P. knowlesi*, strong evidence is lacking for more than half of the entomological surveillance indicators (Table 1). Therefore, quantification of vector control target behaviours like biting location and peak biting times, as well as risk assessment of zoonotic malaria transmission to humans, remains challenging and should be a key focal point of future research.

## Distribution

**Fig. 2:**
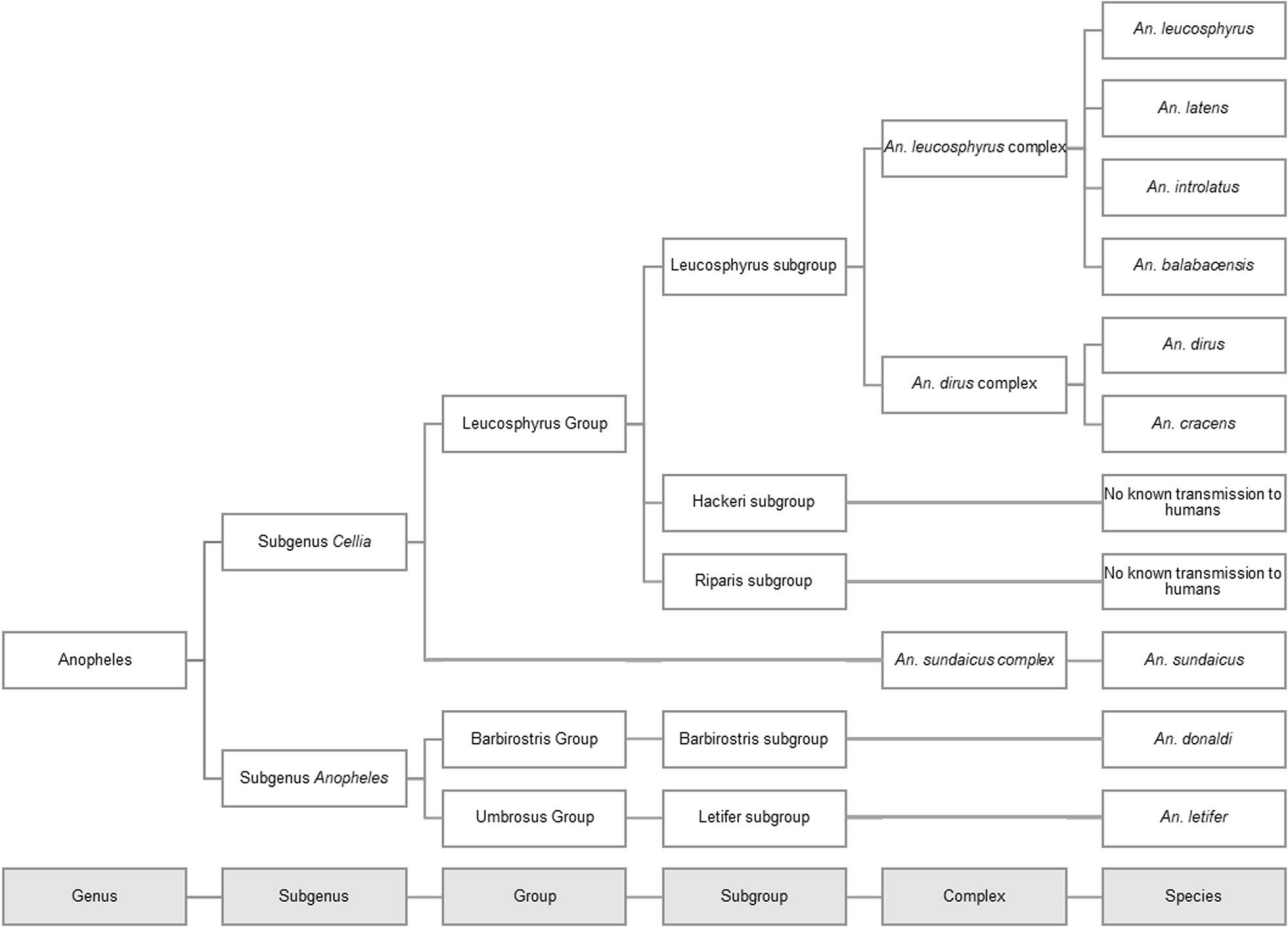
Overview showing the relatedness of all mosquito species that are known or suspected vectors of *Plasmodium knowlesi* to humans

Zoonotic malaria is transmitted by *Anopheles* mosquitoes. In Asia, the main species transmitting *P. knowlesi* and other zoonotic malaria parasites, as well as human-only malaria species in some areas, belong predominantly in the *Anopheles* Leucosphyrus Group [34–36] (see Table 1, Fig. 2). These species are found across a wide geographic range, stretching from Northeast India and Myanmar eastward to Indonesia and the Philippines [37]. The Leucosphyrus Group contains 21 species in three subgroups (Leucosphyrus, Hackeri and Riparis) [34]. The Leucosphyrus subgroup is of most interest from a public health perspective as many species are incriminated as *P. knowlesi* vectors. The Leucosphyrus subgroup contains thirteen species, of which twelve belong in two cryptic species complexes: *An. leucosphyrus* and *Anopheles dirus* [34, 35]. The *An. dirus* complex is the most biodiverse and contains eight known species, all of which occur in continental Southeast Asia [38], with *Anopheles cracens* also found on Sumatra as well as in peninsular Thailand and Malaysia [35]. The four known member species of the *An. leucosphyrus* complex are found in southern Thailand, Malaysia, Indonesia, and the Philippines [39].

Interestingly, the distribution of the Leucosphyrus Group mosquitoes roughly matches the distribution of the long-tailed macaque (*M. fascicularis*). Moreover, the distribution of the *An. leucosphyrus* complex overlaps that of the Southern pig-tailed macaque (*M. nemestrina*) [39]. In addition, the distribution of the *An. dirus* complex closely matches the distribution of the Northern pig-tailed macaque (*M. leonina*). The *An. dirus* complex is bound to continental Southeast Asia (except *An. cracens*, which is found in North Sumatra as well [35]), while the *An. leucosphyrus* complex has a smaller geographical distribution [37].

Recently, studies in Sarawak and Sabah, East Malaysia, incriminated two species not in the Leucosphyrus Group, *Anopheles letifer* [40] and *Anopheles donaldi* [41, 42], as *P. knowlesi* vectors in these regions. The mosquitoes were collected biting humans in both Sarawak and Sabah, and *P. knowlesi* was detected by PCR in the salivary glands and the whole mosquito, respectively. However, sporozoite or oocyst presence was not confirmed by microscopy of the salivary glands or midgut, and salivary glands were only examined separately by PCR in one study. Therefore, vector status of *An. donaldi* and *An. letifer* awaits official confirmation. Additionally, *P. knowlesi* DNA was found in a small pool of *Anopheles sundaicus* in the Nicobar district, India [43]. However, no sporozoites were found and the study was too small to confirm the vector status of *An. sundaicus* for *P. knowlesi*. Further research is required to determine whether *An. sundaicus* may be a vector for *P. knowlesi* to humans. Still, the detection of parasites represents a remarkable exception to the widely accepted paradigm that *P. knowlesi* and *P. cynomolgi* zoonotic malaria are only transmitted by Leucosphyrus Group mosquitoes. It raises the possibility that other anopheline species may be capable of transmitting simian *Plasmodium* species to humans. Hence, public health officials and researchers of zoonotic *Plasmodium* species need to consider more comprehensive studies of anopheline species.

## Vector behaviour

Species within the *An. dirus* and *An. leucosphyrus* complexes are historically considered to be forest and forest fringe dwelling species [34, 35, 37, 44]. The species for which moderate or strong evidence exists for their *P. knowlesi* vector status and behaviours (Table 1) feed predominantly outdoors on both humans and other animals, including macaques. Indoor human biting has been observed; however, these observations have indicated low densities for *An. balabacensis* in Sabah [45] and *An. latens* in Sarawak [46], Malaysia, and higher densities for *An. dirus* in Vietnam [47] and Lao PDR [48]. In these studies, *An. balabacensis* and *An. latens* were collected in houses and a longhouse, respectively. However, *An. dirus* was only collected inside open-walled farm huts or houses devoid of proper walls, leaving the question unresolved of whether *An. dirus* will enter more permanently enclosed houses to feed. Biting occurs generally early in the evening, between 18.00 and 21.00, with sustained low biting rates throughout the night [45, 46, 49–52], although with some recently observed exceptions (Table 2).

Field studies investigating host blood meal choice in Leucosphyrus Group mosquitoes showed highly opportunistic biting behaviour. *Anopheles latens* feeds on humans in the forest fringe and both humans and macaques in the forest in Sarawak, East Malaysia [46]. Additionally, biting behaviour may also depend on height above the ground in the forest canopy. Height-dependent biting behaviour in *An. leucosphyrus* complex mosquitoes was shown by Harbach et al*.* in the 1980s in South Kalimantan [49], with a higher human biting rate in the forest canopy than on the ground. Another field study in Sarawak, comparing the human landing catch with a monkey-baited trap, showed that *An. latens* fed more on macaques than humans in the canopy but fed more on humans than macaques near the ground [50]. A similar pattern was noted in Peninsular Malaysia, for *An. leucosphyrus* [30] and for *Anopheles introlatus* (formerly *An. balabacencis introlatus*) [53]. In Sabah, Malaysian Borneo, *An. balabacensis* bites humans more at ground level than in the canopy during paired human landing catches 55]. However, no direct comparisons to macaques were made, so it remains unclear if potential vectors were diverted to macaque hosts in the canopy [54]. Still, these observations are consistent with the overall pattern observed for the *An. leucosphyrus* complex. Interestingly, *An. dirus* in Cambodia was shown to preferentially bite humans on the ground and macaques in the canopy [55]. This regularly observed propensity to feed on both humans and macaques in forests, forest fringes and fragmented forest habitats means that these mosquitoes can act as bridge vectors to transmit simian malaria species to humans.

Several suspected *P. knowlesi* vector species remain severely understudied. *Anopheles introlatus*, a potential vector in Peninsular Malaysia, bites both humans and macaques [53]. Although *P. knowlesi* oocytes have been detected in 2014 [56], evidence that *An. introlatus* can develop a sporozoite stage infection of *P. knowlesi* was found very recently [57] (unpublished data, Vythilingam, UM). This is contrary to *An. donaldi* and *An. letifer*, for which there exists some molecular evidence [40, 42]. Additionally, *P. inui* and *Plasmodium fieldi* sporozoites, two other simian *Plasmodium* species which might be transmitted to humans, were detected in *An. introlatus* and *An. cracens* [58]. It is known from Vietnam that, when *An. dirus* complex mosquitoes can develop sporozoites for one simian malaria species, they have the ability to develop sporozoites for all other species [52, 59]. This implies that *An. leucosphyrus* Group mosquitoes are able to develop all five species of simian malaria (*P. knowlesi, P. cynomolgi, P. inui, P. coatneyi, P. fieldi*). Additionally, almost nothing is known about the host preference, feeding habits or infection rates of *An. leucosphyrus*. This species is suspected to be confined to Sumatra [60] and further research is required to ascertain whether this species may transmit *P. knowlesi* to humans to guide appropriate vector control.

All species from the *An. leucosphyrus* complex, except for *An. leucosphyrus*, have been found carrying *P. knowlesi* sporozoites and hence have the potential of transmitting *P. knowlesi* malaria to humans [50, 51, 56]. However, only two species from the *An. dirus* complex were found positive for *P. knowlesi* sporozoites. These are *An. dirus* in Vietnam [21, 61], and *An. cracens* [62] in Peninsular Malaysia. Of these, *An. dirus* is of most concern due to its wide distribution across multiple Southeast Asian countries and the high numbers in which it often occurs [63, 64]. Although infected *An. dirus* were only found in South-Central Vietnam [21, 65], the similarity of its distribution with that of long-tailed and pig-tailed macaques and its opportunistic blood feeding behaviour could make it a highly probable vector [39]. Especially people who stay overnight in forest or forest fringe areas where *An. dirus* is present are at risk of infection with *P. knowlesi* [52]. The range of *An. cracens* comprises areas of Peninsular Malaysia and Sumatra [35], where it has been incriminated as an important vector of human *P. knowlesi* infections in the former area [12, 66].

## Larval habitats

All members of the Leucosphyrus Group are essentially forest mosquitoes, and their larval habitats reflect this. However, recent research found associations between *An. balabacensis* larval habitats, distance from (rubber) plantations and forest fragmentation, supporting the hypothesis that the vector has adapted to changing land-use patterns 68]. Although larval habitat documentation is sparse (see Table 1), there is much overlap among the larval habitat preferences of the studied species (*An. dirus *sensu lato (*s.l*.), *An. leucosphyrus s.l*., *An. balabacensis*) [34, 35]. Larvae are mostly found in freshwater pools and puddles that are often temporary (Table 2). These water bodies can originate from almost any source after sufficient rainfall, including elephant footprints and wheel tracks to larger puddles on the ground [68]. Common characteristics of these habitats are that they are, at least partially, shaded and that the water is still [34, 37], with little to no vegetation present. Larvae can occur in large densities (MJ Bangs, pers. comm.), which leads to the assumption that these species are most abundant during the rainy season or after a prolonged period of precipitation during the dry season. *Anopheles leucosphyrus* complex mosquitoes seem to prefer temporary water bodies rather than more permanent water bodies like streams, while *An. dirus* can also be found along streams when the current is slow [38, 69].

## Drivers for transmission

Zoonotic malaria was traditionally considered a ‘forest malaria’ with infections mainly in people who enter the forest for work, like loggers or hunters [21, 59]. However, expansion of human activities and the resulting fragmentation of forests in large parts of Southeast Asia has been associated with increasing numbers of zoonotic *Plasmodium* species infections, not only in forest workers, but notably also in agricultural workers who remain relatively close to their resident village [70–73]. Zoonotic infection spill-over is notoriously hard to predict, as it often crosses various phylogenetic and spatiotemporal scales [74]. The behaviour of the monkeys, mosquitoes and people influences their interactions with each other, all of which are heterogeneous in space and time. One of the most important drivers of zoonotic malaria spill-over to humans is the ecology of mosquito vectors and reservoir hosts [75]. To become infected with zoonotic *Plasmodium* species, infectious mosquitoes must bite humans. This requires proximity to infectious vectors, which is often associated with changes in land-use, occupation, and house construction [76]. Additionally, wildlife harbouring the parasites (the reservoir hosts) needs to be close to both humans and vectors that readily blood-feed on both humans and the reservoir species. *P. knowlesi* (as well as *P. cynomolgi*) usually results in benign, chronic infections in natural macaque hosts [5, 77]. Hence, infected monkeys form an ideal reservoir for parasite spill over to humans as the monkeys are not restricted in their normal behaviours by disease [5]. As the interaction between monkeys, mosquitoes and humans influences their respective behaviours, the factors discussed in the following subsections are in fact closely connected and interacting.

## Vector diversity

The large number of sympatric species in Southeast Asia that can transmit malaria, including zoonotic malarias, makes it hard to determine the dominant vector in a geographic region. Variation in behaviours and distributions of individual vectors associated with a high diversity of vector species are likely to affect the transmission dynamics of zoonotic malaria, especially when vector abundance increases [78]. Although this depends on the competence of the present vector species, a higher number of sympatric vector species in an area will generally facilitate increased zoonotic malaria transmission [79]. Hawkes et al. [41] observed increased *Anopheles* species richness and abundance, as well as a higher infection rate, in forest edges compared to human settlements and plantations. Higher species richness can also extend the duration of seasonality in pathogen transmission, thus enabling a longer period of mosquito biting activity [80]. In addition, the high degree of behavioural plasticity observed in many species plays an important role in the large variation in dominance of different vector species [81]. This variation makes targeting zoonotic malaria vectors challenging, because species can display different behaviours by geographic area [38].

Human activities can drastically change the community composition of both vector and reservoir species. Anthropogenic exploitation of natural resources, like logging or hunting, in addition to the expansion of human settlements and, to a lesser extent, plantations, can cause a general loss of biodiversity [82, 83]. As a result of this development, the vector community composition can change, as has been observed in Kinabatangan, Malaysian Borneo, where *An. donaldi* may have replaced *An. balabacensis* as the primary malaria vector in certain areas [84]. In Sarawak, Malaysian Borneo, *An. donaldi* and other suspected malaria vector abundance decreased while *Aedes albopictus* numbers increased after anthropogenic disturbance, thereby contributing to a shift in the relative disease risk from malaria to arboviruses [85]. Additionally, if biodiversity loss is more severe in vertebrates than in invertebrates [86], an amplification effect for pathogen transmission can occur. Hence, high vector species richness concentrates blood feeding on the limited vertebrate species, resulting in higher biting on reservoir species. However, research is required to clarify the exact implications of this theoretical mechanism for zoonotic malaria and, specifically, *P. knowlesi* transmission.

## Host diversity and distribution

As the primary hosts and vectors of *P. knowlesi* and *P. cynomolgi* are originally forest-dwelling species, contacts between humans, macaques and mosquitoes were few and transmission was thought to be very rare [30, 53]. However, human activities can lead to provision (unintentional or intentional feeding) of macaque troops [87], thus eventually drawing potential carriers of zoonotic malaria species towards areas of human settlement. The macaques are highly invasive and readily adapt their behaviour to thrive in fragmented landscapes by raiding crops, exhibiting aggressive behaviour and becoming an urban nuisance [88]. Macaque behaviour is disturbed by deforestation, and changes have been observed in the macaque troop home range size, movement speeds and use of different habitat types [87, 88]. Long-tailed and pig-tailed macaques are frugivores but will switch readily to other, more abundant food sources in the absence of fruits [89]. Pig-tailed macaques in Peninsular Malaysia extended their home range significantly to forage in oil palm plantations, which provided them with abundant, year-round food sources [90]. Food provision in the vicinity of human settlements likely causes macaque troops to remain around these settlements. Ruslin et al. [91] showed that long-tailed macaques will readily feed on anthropogenic food and food waste. Additionally, Stark et al. [88] suggested that long-tailed macaques in Sabah, East Malaysia actively avoid human logging activities, spending more time in other habitats including farmland and thus bringing the *P. knowlesi* reservoir closer to humans.

Changes in biodiversity have the potential to affect the risk of infectious disease emergence [92]. If vectors can select bloodmeals from a variety of host species that differ in their reservoir competence, the probability of a parasite being transmitted from host to vector will be diminished. The presence of low-capacity hosts (incompetent reservoirs) has been hypothesized to dilute the effect of the highly competent reservoir hosts, thus reducing disease risk, and is termed the dilution effect [92].

## Environmental change

The emergence of zoonotic malaria in Southeast Asia is thought to be strongly driven by environmental changes caused by humans. When the first large focus of *P. knowlesi* malaria was discovered in 2004 [8], it was hypothesized that *P. knowlesi* infections were contracted away from human settlements in the forest. However, later research in the same region revealed that infective *An. latens*, the dominant vector species in the region, preferred to bite humans in farm areas and forest fringes [46]. A similar pattern was observed in Sabah, where the predominant *P. knowlesi* vector, *An. balabacensis*, had the highest abundance in villages but the highest vectorial capacity in farms and forest fringes, based on parous rate and life expectancy [51]. In mainland Southeast Asia, the dominant vector species *An. dirus* occurred in high densities in the forest rather than forest fringes or villages, but human invasion and sustained activities in the forests exposed people to infectious bites [38, 39, 45]. Indeed, increased human activities in the forest may increase vector density in the forest and forest edge, relative to the village, by both creating more oviposition sites through human activities (e.g., puddles in muddy roads) [67] and by providing more human blood hosts [68].

The strongest environmental driver of *P. knowlesi* infections is the fragmentation of forests resulting from anthropogenic land-use [93]. Fornace et al. [94] found that the decline of forest cover, both recent and historical, in the vicinity of human settlements was associated with a greater *P. knowlesi* incidence in Sabah, Malaysia. In addition, when human land-use and movements during peak biting times were considered, the highest risk of exposure to infectious mosquito bites was found in forest fringes, rather than the forest where higher vector abundance was observed [95]. More specifically, factors that increase *P. knowlesi* infection risk were all associated with increased human activities in forest fringes or disturbed, fragmented forests [70]. Occupation has been a consistent major risk factor, with oil palm plantation work and subsistence farmers having a higher exposure risk [70]. The aforementioned anthropogenic land-use changes can affect the transmission of zoonotic malaria to individuals as well as populations. Besides when humans enter the forest (i.e., for work) and thus the zoonotic cycle, the removal of intact forest corridors can force the macaque reservoir hosts of zoonotic malaria into human territory, after which the mosquito vectors will most likely follow (with transmission to the human population) ([96]. Additionally, the replacement of primary forest with farms or plantations, notably large-scale oil palm, causes significant biodiversity loss in both vertebrates and, to a slightly lesser extent, invertebrates [97, 98]. The adaptation of *P. knowlesi* reservoir hosts and vectors to human habituation, combined with the loss of any dilution effect, can further increase the exposure risk of humans [99]. It is important to keep in mind that the knowledge base is too limited to inform on possible mechanisms that regulate zoonotic malaria vector behaviours and how anthropogenic land-use changes might influence these behaviours.

## Conclusion

Current information on zoonotic malaria vector species is largely based on a limited number of studies in geographically restricted areas (predominantly in Malaysia). The dearth of information on key vector behaviours stands in the way of effective vector control, especially considering the strong increase in zoonotic malaria infections in the past decade. Additional data is particularly needed from currently understudied regions where previously incriminated or suspected zoonotic malaria vectors occur. The way that humans change the environment results in increased exposure to simian malaria species and could facilitate vector adaptation to humans. However, how changing human land-use influences zoonotic malaria vectors is still poorly understood. Hence, more data are needed on vector diversity and bionomics in relation to anthropogenic land-use change. Understanding the individual vectors involved in zoonotic malaria transmission and the variation in their behaviour is imperative to deploy effective mosquito control methods, which remain key to reducing the malaria burden.

## Data Availability

Data sharing is not applicable to this article as no datasets were generated or analysed during the current study.
